# Genome-informed investigation of the molecular evolution and genetic reassortment of severe fever with thrombocytopenia syndrome virus

**DOI:** 10.1371/journal.pntd.0011630

**Published:** 2023-09-15

**Authors:** Kyuyoung Lee, Jong Hyeon Seok, Hyunbeen Kim, Sejik Park, Sohyun Lee, Joon-Yong Bae, Kyeongseok Jeon, Jun-Gu Kang, Jeong Rae Yoo, Sang Taek Heo, Nam-Hyuk Cho, Keun Hwa Lee, Kisoon Kim, Man-Seong Park, Jin Il Kim

**Affiliations:** 1 Department of Microbiology, Institute for Viral Diseases, College of Medicine, Korea University, Seoul, Republic of Korea; 2 Department of Microbiology and Immunology, College of Medicine, Seoul National University, Seoul, Republic of Korea; 3 Laboratory for Vector Borne Disease, Korea Zoonosis Research Institute, Jeonbuk National University, Iksan, Republic of Korea; 4 Department of Internal Medicine, College of Medicine, Jeju National University, Jeju, Republic of Korea; 5 Department of Microbiology, College of Medicine, Hanyang University, Seoul, Republic of Korea; 6 Vaccine Innovation Center, College of Medicine, Korea University, Seoul, Republic of Korea; 7 Biosafety Center, College of Medicine, Korea University, Seoul, Republic of Korea; George Washington University, UNITED STATES

## Abstract

**Background:**

Severe fever with thrombocytopenia syndrome virus (SFTSV) is a viral pathogen causing significant clinical signs from mild fever with thrombocytopenia to severe hemorrhages. World Health Organization has paid special attention to the dramatic increase in human SFTS cases in China, Japan, and South Korea since the 2010s. The present study investigated the molecular evolution and genetic reassortment of SFTSVs using complete genomic sequences.

**Methods/Principal finding:**

We collected the complete genome sequences of SFTSVs globally isolated until 2019 (L segment, n = 307; M segment, n = 326; and S segment, n = 564) and evaluated the evolutionary profiles of SFTSVs based on phylogenetic and molecular selection pressure analyses. By employing a time-scaled Bayesian inference method, we found the geographical heterogeneity of dominant SFTSV genotypes in China, Japan, and South Korea around several centuries before and locally spread by tick-born spillover with infrequent long-distance transmission. Purifying selection predominated the molecular evolution of SFTSVs with limited gene reassortment and fixed substitution, but almost all three gene segments appeared to harbor at least one amino acid residue under positive selection. Specifically, the nonstructural protein and glycoprotein (Gn/Gc) genes were preferential selective targets, and the Gn region retained the highest number of positively selected residues.

**Conclusion/Significance:**

Here, the large-scale genomic analyses of SFTSVs improved prior knowledge of how this virus emerged and evolved in China, Japan, and South Korea. Our results highlight the importance of SFTSV surveillance in both human and non-human reservoirs at the molecular level to fight against fatal human infection with the virus.

## Author summary

SFTSV causes a high mortality disease in humans, especially in China, Japan, and South Korea. For a better understanding of the evolutionary characteristics of SFTSVs, our study performed phylogenetic and genetic reassortment analyses using the largely collected genomic information of SFTSVs. It appeared that dominant endemic SFTSVs in China, Japan, and South Korea likely diverged several centuries ago. Movement of vector (e.g., tick) or non-human mammalian reservoirs possibly played a key role in human SFTSV spillover and infrequently led to international spread among three countries. Purifying selection strongly limited the generation of novel variants through either molecular mutations or genetic reassortment. However, we observed a few amino acid sites under positive selection, especially in glycoproteins which might limitedly allow the generation of variants within a clade, in which molecular evolution might not be a major cause for the current emergence of SFTSVs but the ecological or epidemiological change possibly be a more plausible factor. However, we should still carefully monitor the evolutionary dynamics of SFTSV considering the potential of host adaptation in humans.

## Introduction

Severe fever with thrombocytopenia syndrome (SFTS) was first reported from hospitalized patients in China in 2009, and its causative virus (SFTSV) was identified in 2011 [1]. The SFTSV is one of the viruses belonging to the genus *Phlebovirus* of the family *Phenuiviridae* in the Order *Bunyavirales* [2]. It retains single-stranded, negative-sense RNA genome segments (L, M, and S) [1]. The L gene encodes an RNA-dependent RNA polymerase (RdRp) for viral genome replication and transcription [3], the M gene encodes glycoproteins N (Gn) and C (Gc) for viral attachment and entry to host cells [4], and the S gene encodes nucleocapsid (NP) and nonstructural (NS) proteins for viral replication protection and immune evasion [5,6].

**Fig 1:**
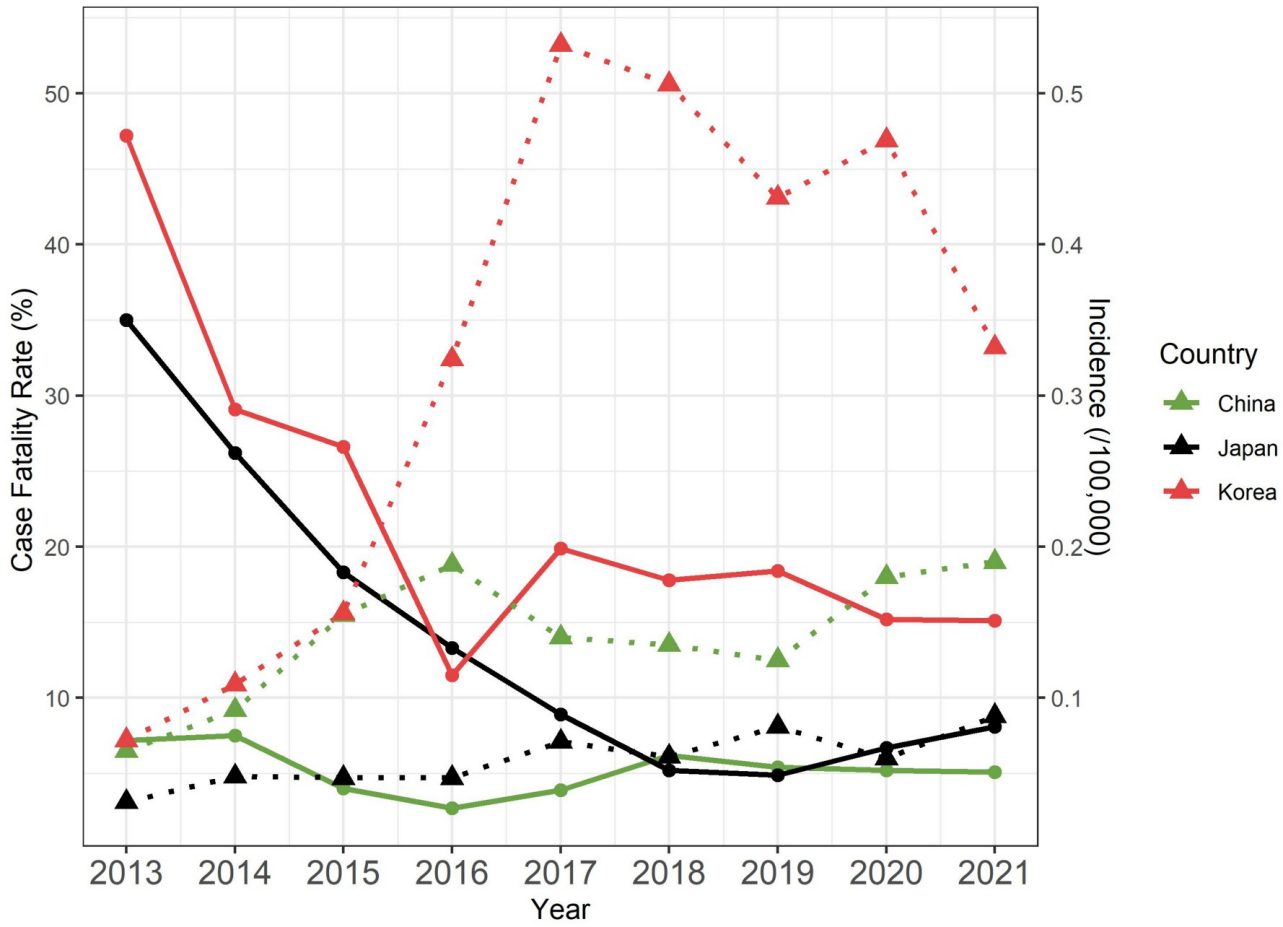
Summary of incidence and case fatality rate (CFR) of human SFTSV infection from 2013 to 2021. Annual incidence (Dotted and triangle) and CFR (Line and circle) of the SFTSV in China (Green), Japan (Black), and South Korea (Red) from 2013 to 2021. The plot was reconstructed from the statistics of national surveillance reports in China [15], Japan [16], and South Korea [17].

Since the first identification of SFTSV, the number of human infection cases with the virus drastically increased in China [7,8], as well as in South Korea [9], Japan [10,11], and Vietnam [12], and more than 13 thousand human cases were reported globally until 2019 [7,8]. SFTSV causes clinical symptoms in humans from mild fever with thrombocytopenia and leukopenia to severe hemorrhages, encephalitis, or multiple organ failures to death [1,13]. The case fatality rate (CFR) of SFTS showed differences by epidemiological and environmental factors but remained quite high in China (2.7–8.0%), Japan (4.9–35.0%), and South Korea (11.5–47.2%) (Fig 1) [7–10,14–17].

Considering its clinical and epidemiological significance in public health, the World Health Organization (WHO) listed the virus as one of the most threatening pathogens with a strong potential for epidemic [13]. Current surveillance presumed that the incidence of human SFTS could increase, and the geographic distribution of human infection would be expanded further over time [8,18,19]. Tick bite, specifically by *Haemaphysalis longicornis*, is a well-known transmission factor of SFTS in humans considering a broad host range, geographical distribution, and virus isolation [20–22]. Previous investigations supported that human SFTSV infection might be incidental spillover events from viral reproduction in the life cycle of the tick or the transmission cycle of the tick with sub-clinically infected animals [23–26].

Many studies investigated the genetic diversity, molecular evolution, and transmission of SFTSVs across the three most affected countries: China, Japan, and South Korea [9,11,27–29]. These studies generally collected genomic information from SFTSV isolates and performed a phylogenetic analysis to classify their SFTSVs into five genotypes and to detect key mutations [11,27,29,30]. One study reconstructed epidemiological circumstances among China, Japan, and South Korea from the genotypic relatedness of SFTSV [27]. However, many studies likely showed a potential for a sampling bias in phylogenetic inferences considering the limited number of genomic information. Here, we revisited the phylogenetic and evolutionary analyses of SFTSVs using the large-scale genomic sequence data compared to previous research. We evaluated the genetic characteristics of the three gene segments of SFTSVs using phylogenetic approaches. Furthermore, our study investigated the genetic reassortment and selection pressure profiles of the three gene segments at the molecular level to better understand the molecular evolutionary dynamics of SFTSVs.

## Materials and methods

### Ethics statement

The present study used the genomic sequences of SFTSV from human clinical specimens regarding the institutional review board (IRB) approved at Jeju National University Hospital (IRB file no. 2015-08-002). The formal statements of written consent were obtained from the all the participants.

### Data collection

Our study collected nucleotide sequences of L (6,252 nucleotides, nts), M (3,222 nts), and S (1,744 nts) gene segments of SFTSVs from the database of the Bacterial and Viral Bioinformatics Resource Center (BV-BRC) (https://www.bv-brc.org/). Epidemiological information about collection dates, countries, and host species were obtained based on the accession number of the genome sequences. The present study additionally involved genomic sequences of SFTSVs during the outbreak in Japan from 2013 to 2014 provided by the previous phylogenetic study (L, n = 38; M, n = 35; and S, n = 56) [30]. We also used the genomic sequences of SFTSV isolated from human clinical specimens collected (L, n = 8; M, n = 8; and S, n = 34) and ticks captured in South Korea (L, n = 1; M, n = 1; and S, n = 3). Our study excluded gene sequences if a sample did not have full length of nucleotide sequences, nor provide collection dates, countries, and host species. Furthermore, we only involved one representative sequence among multiple sequences with very high nucleotide sequence identity if the sequences were collected from the same date, country, and host species. Finally, a total of 1,197 genome sequences (L, n = 307; M, n = 326; and S, n = 564) of SFTSVs were analyzed in this study (S1 Table and S1 Data). Each sequence set was aligned using Multiple Alignment using Fast Fourier Transform approach (version 7), [31] and manually adjusted (S1 Data).

### Phylogenetic analysis

Bayesian Markov chain Monte Carlo (MCMC) Metropolis–Hastings algorithm was used for the phylogeny estimation of the three gene segments (overall sequence sets for the L, M, and S genes) on BEAST (version 1.10.4, [32]). Our study used jModelTest2 (version 2.1.10) to select the best-fit model for Bayesian phylogeny estimation for the three gene segments through the phylogenetic model averaging [33]. We selected General Time Reversal (GTR) + I + Γ model [34] as the nucleotide substitution model with uncorrelated relaxed, lognormal clock model and Bayesian skygrid tree prior for the analysis. Our study initiated the MCMC chain from 50 million runs with a 10% burn-in and sub-sampled in every 50 thousand iterations. If the MCMC chain showed the lack of convergence with low effective sample sizes, we performed the Bayesian estimation with higher MCMC chain runs and repeated this step until the MCMC chains reached to appropriate convergence, stationary, and mixing properties with the sufficient effective sample sizes (>100) on Tracer (version 1.6) [35]. The maximum clade credibility (MCC) trees of the three gene segments were summarized using TreeAnnotator (version 1.10.4) and visualized by FigTree (version 1.4.2). The Bayesian phylogeny was used to estimate the evolutionary rates and the time of the most recent common ancestor (tMRCA) of the three gene segments of SFTSVs in four datasets. Estimates of the evolutionary rate and tMRCA were presented as median along with the lower and upper limits of the 95% highest probability density (HPD). All statistical estimates of phylogenetic analysis were summarized and visualized using “ggplot2” package on R (version 4.1.3) [36] with R studio (version 2022.12.0) [37].

### Reassortment analysis

Our study detected reassortment events in each pair of gene segments using the graph incompatibility-based reassortment finder (GiRaF) approach (version 1.2) [38]. Alignments of the L, M, and S gene segments in the full dataset were used for the reconstruction of phylogenetic relationship in MrBayes (GTR+I+Γ, 1,000,000 iterations, 10% burn-in, and sampling every 10,000 iterations) [39]. The phylogenetic uncertainty was statistically estimated by the trees of three gene segments in the GiRaF. We performed three independent GiRaF trials in each pair of gene segments and confirmed reassortment events exhibited in all three trials.

### Natural selection pressure profile

Natural selection pressure profiles were measured by the functional subunit of coding regions in the three gene segments of SFTSVs (L: RdRp and C-terminus regions; M: Gn and Gc regions; and S: NP and NS regions). The selection pressure was estimated by the ratio between nonsynonymous (dN) and synonymous substitutions rates (dS) employing the best-fit REV nucleotide substitution model (cutoff *p*-value <0.05). The dN/dS ratios were estimated by four methods, the single likelihood ancestor counting (SLAC) [40], fast unconstrained Bayesian approximation (FUBAR) [41] (cutoff posterior probability > 0.9), mixed effects model of evolution (MEME) [42] (cutoff *p*-value <0.1) in the HyPhy (version 2.2) on the Datamonkey web-server (http://www.datamonkey.org, [43]) and the Renaissance counting (RNSC) method implemented in BEAST (cutoff *p*-value <0.1) [44].

## Results

### Phylogenetic relationships of the SFTSV gene segments

**Fig 2:**
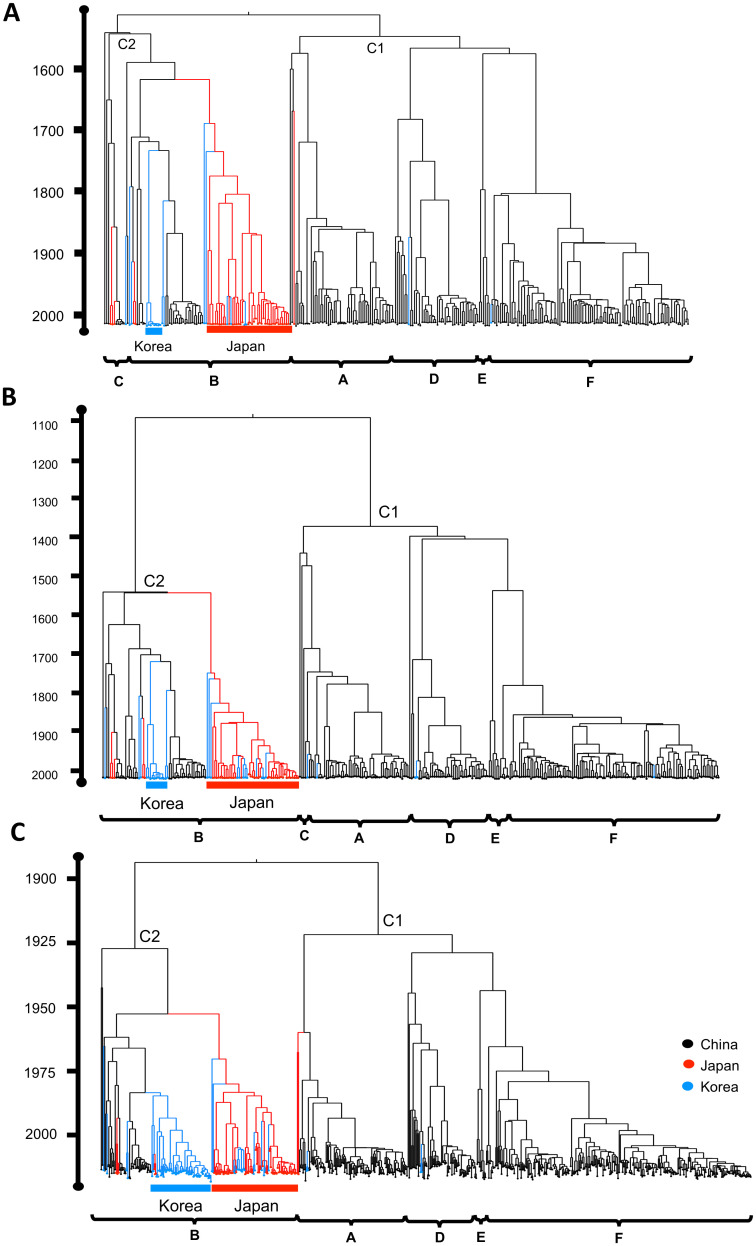
Phylogenetic relationships of SFTSV. The maximum clade credibility tree of the (A) Long (L), (B) Medium (M), (C) Short (S) gene segments was reconstructed using the Bayesian evolutionary interference method. The brackets in the x-axis display the genotype classification by Fu et al (2016). The phylogenetic branches with specific countries were color labeled black (China), red (Japan), and blue (South Korea), respectively.

All phylogenetic trees of L, M, and S genes in SFTSVs showed highly homogeneous topology comprising co-circulation of multiple lineages with intra-subtype diversification (Fig 2). Following the genetic clade definition by Fu et al. [27], we observed two major clades, Clade 1 (genotypes A, D, E, and F) and Clade 2 (genotype B) of SFTSVs. The reference strain of genotype C (China/AHL/2011; GenBank accession number JQ670934) showed incongruence of classification between the L (Clade 2) and M (Clade 1) genes, and its S gene belonged to genotype D. Most Chinese SFTSVs formed multiple genotypes in Clade 1, and only a few strains belonged to genotypes B and C (or genotype B) in Clade 2. Japanese SFTSVs formed one subclade in genotype B in Clade 2. Only a few strains belonged to either other subclades in genotype B or genotype A in Clade 1. Most South Korean SFTSVs formed subclades in genotype B, and a few minor strains belonged to either Chinese or Japanese subclades in genotype B, or rarely in Clade 1 (each of A, D, or F genotype). On the other hand, the phylogenetic locations of different host SFTSVs (human, tick, and non-human mammal hosts) displayed a strong polyphyletic pattern similarly in all three gene segments (S1 Fig). The SFTSVs isolated in tick and non-human mammal hosts were reported in China and South Korea in both Clades 1 and 2 and showed genetic relatedness with human SFTSVs in all three gene segments.

### Evolutionary profiles and genetic reassortment of the SFTSV gene segments

**Fig 3:**
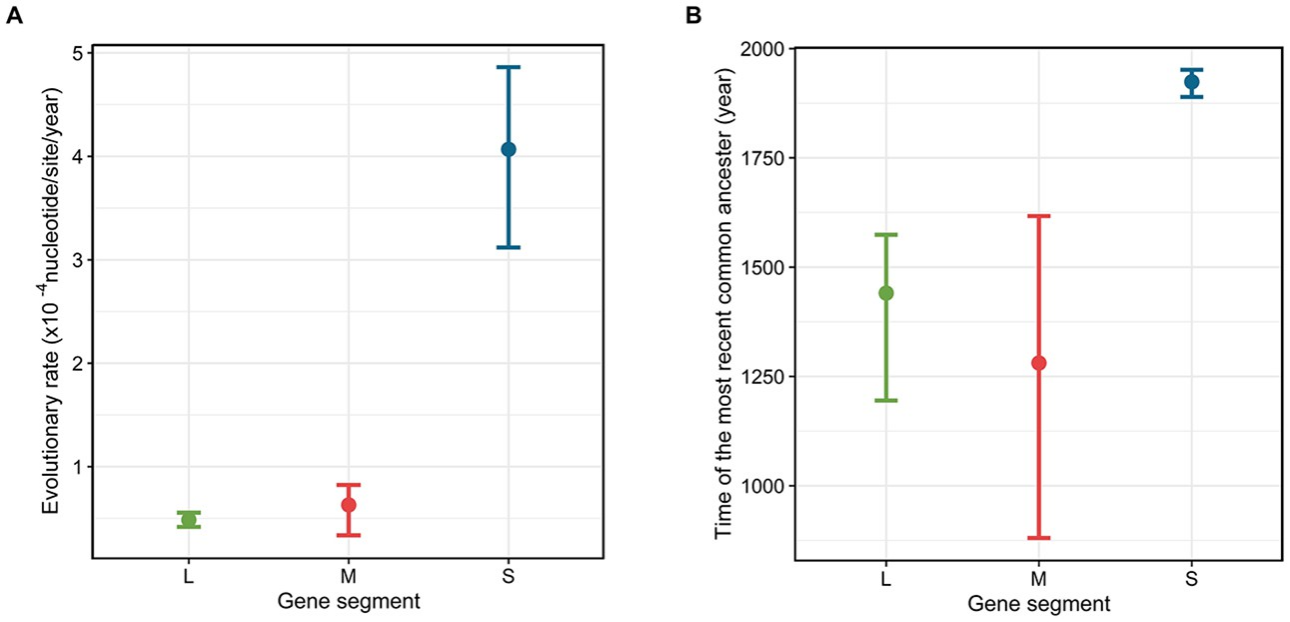
Evolutionary rate and the time of most recent common ancestor (tMRCA) of three gene segments in SFTSV by three countries. (A) The Evolutionary rate and (B) tMRCA of Long (L), Medium (M), Short (S) gene segments were summarized by median and 95% HPD.

SFTSVs exhibited different evolutionary rates and tMRCAs by three gene segments even with similar phylogenic genealogy (Fig 3). The S gene segment showed the highest evolutionary rate (4.06 x 10^−4^ substitutions/site/year) among the three gene segments, which was approximately 2 to 19 times higher than that of the other two genes (Table 1). As the differences in the evolutionary rates among the three gene segments, the S gene showed the youngest tMRCA than the other two genes (Table 1).

**Table 1:** The estimates of the evolutionary rate and the time to the most recent common ancestor (tMRCA) in the phylogenetic analysis for SFTSV.

Gene segment	Evolutionary rate (x10^-4^ nucleotides/site/year) (95% HPD)	tMRCA (Year) (95% HPD)
L	0.486 (0.419,0.556)	1424.05 (1194.92,1574.14)
M	0.636 (0.337,0.823)	1250.47 (880.66,1616.86)
S	4.069 (3.119,4.862)	1921.09 (1889.3,1951.35)

**Fig 4:**
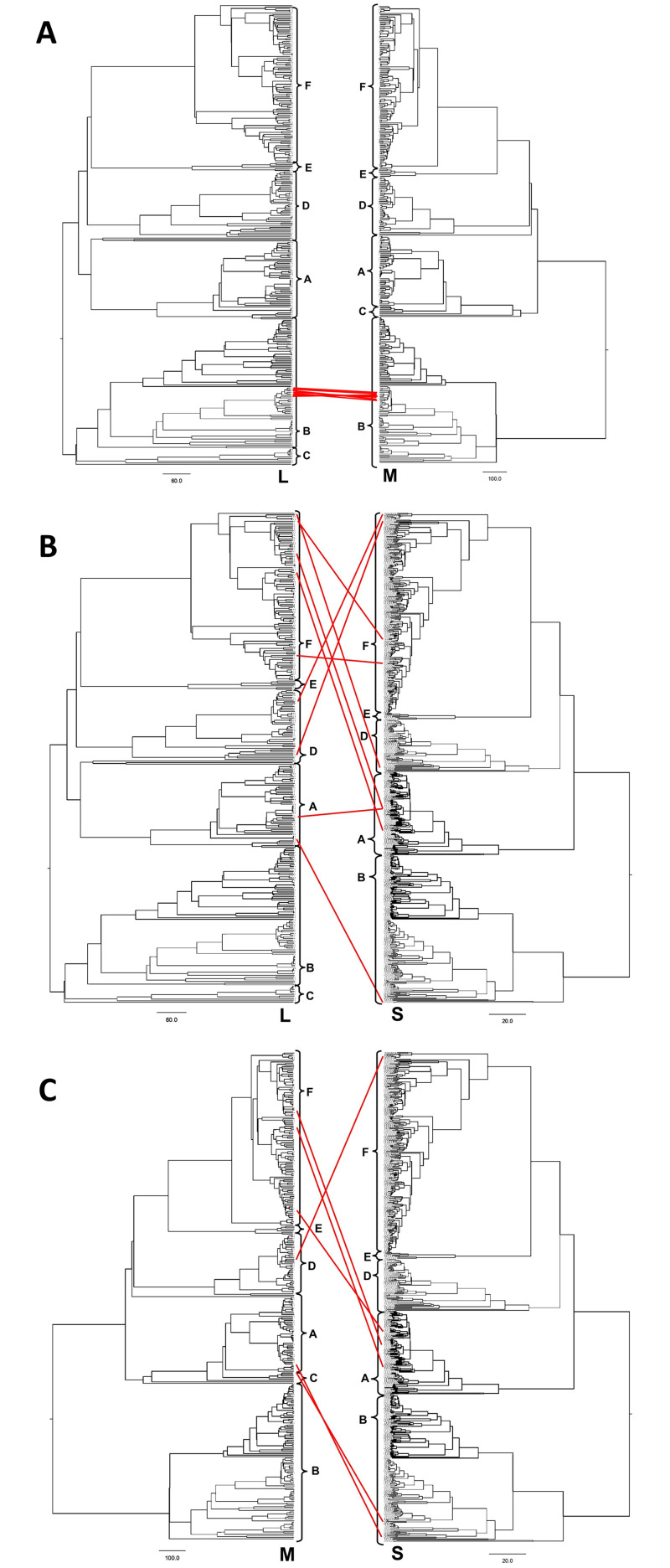
Reassortment between pairs of three gene segments in SFTSV. (A) Reassortment of the L and M gene, (B) the L and S gene (C) the M and S gene segments. The red line between phylogeny depicted gene reassortment between two genes. The brackets in the y-axis display the genotype classification by Fu et al (2016).

Our study observed 22 genetic reassortment events between the three gene segments in 18 SFTSVs (Fig 4 and S2 Table). All reassortment events were detected only in Chinese SFTSVs sampled from humans between 2011 to 2016. The reassortment events between the L and M gene segments (L-M reassortant) were exclusively observed in one subclade belonging to genotype B in Clade 2 (Fig 4A). While the 8 L-M reassortant strains switched their gene segments from the neighboring subclade, two functional subdomains in the L gene did not show any amino acid substitution. However, the Gc domain in the M gene obtained one amino acid substitution (M-Gc: Q297P). The reassortment events of the S gene with the L (L-S reassortant) or M (M-S reassortant) genes were widely observed in both Clades 1 and 2 (Fig 4B and 4C). All M-S reassortant (6/6) and 75% of the L-S reassortant SFTSVs (6/8) switched their S gene segment with SFTSVs belonging to other genotypes. We did not observe any generation of a subclade originating from either L-S or M-S reassortants.

### Natural selection profiles of the SFTSV gene segments

A large fraction of the three gene segments of SFTSVs harbored amino acid residues under purifying selection and showed significant heterogeneity of local selection pressure by genetic regions. All estimates of the global dN/dS ratios in the six protein-coding regions (L gene, RdRp, and C-terminus; M gene, Gn and Gc; and S gene, NP and NS) in the L, M, and S genes ranged from 0.03 to 0.13, which were much lower than 1 (Table 2). Accordingly, the fractions of amino acid sites under statistically significant purifying selection varied from 61.77% (NS in the S gene) to 81.1% (c-terminus of the L gene). However, at least 1 to 5 amino acid residues were found to be under positive selection pressure across the six protein-coding regions. The Gn domain in the M gene had the highest number of amino acid residues (n = 5) under positive selection. The RNSC method revealed that synonymous substitution rates were 3 to 12 times higher than their non-synonymous substitution rates in the three gene segments (Table 2). The NS in the S gene segment showed the highest non-synonymous substitution rate (1.14 x 10^−4^ substitutions/site/year) whereas the NP showed the highest synonymous substitution rate (3.98 x 10^−4^ substitutions/site/year) among all protein-coding domains.

**Table 2:** Molecular selection pressure of the SFTSV genomes.

		Amino acid sites^a^ under positive selection			No. of Negative selection sites ^d^ (%)	dN/dS	Evolutionary rate (10^−4^ substitutions/site/year)	
Gene Segment	coding region	FUBAR^b^	MEME^c^	RNSC^b^			Synonymous	Non-Synonymous
L	RdRp	**2**, **251**	**2**, **251**	**2**, **251**	1004 (77.71%)	0.05	0.223	0.027
	cTerminus	**61**	**61**	**61**	642 (81.06%)	0.04	0.468	0.048
M	Gn	**18**, **151**, **279**, **304**, 476	**18**, 60, **151**, **279**, **304**	**18**, 60, **151**, **279**, **304**	383 (74.22%)	0.09	2.454	0.605
	Gc	n.d.^e^	77, 398	77, 398	341 (66.73%)	0.07	0.486	0.087
S	NP	n.d.	n.d.	n.d.	163 (71.49%)	0.03	3.980	0.324
	NS	**17**, **229**	**17**, **229**, 289	**17**, **229**, 289	181 (61.77%)	0.13	3.705	1.137

^a^ The site under positive selection was predicted by three methods and the numbers describe amino acid sited detected at least two methods are shown, of which those in all three are represented in bold. See S1 Table for the complete list of site numbers.

^b^ Cutoff p-value ≤ 0.1

^c^ Cutoff posterior probability ≥0.95

^d^ The number (percentage) of amino acid sites under negative selection predicted by FUBAR

^e^ n.d., not detected

## Discussion

Since the early 2010s, human infection of SFTSV has been mainly reported in China, Japan, and South Korea. Our phylogenies of three gene segments uniformly depicted that endemic SFTSVs have mainly caused human infection within limited local areas. However, even with the genetic homogeneity of endemic SFTSVs by countries, we occasionally observed polyphyletic genealogy in the phylogenies likely depicting the long-distance spread of endemic SFTSVs among China, Japan, and South Korea [27,30], but not frequent as the phylogenies of influenza virus or SARS-CoV-2 with highly intensive international spread [45,46]. Chinese SFTSV genotype B was most prevalent on Zhoushan Island located in the East China Sea but also caused human infection in South Korea and Japan [27]. Furthermore, Japanese endemic genotype B and genotype A, C, D, and F were detected in human SFTS cases in South Korea. As previous studies uniformly addressed [23–25], the close genetic relatedness of SFTSVs among humans, ticks, and non-human mammals in our study supported that the vector-borne spillover of SFTSVs by ticks would play a key role in human infection from livestock or wildlife reservoirs (S1 Fig). With the limited number of reports of patient-to-patient SFTSV transmission through the direct contact of blood or organ [47,48], long-distance movement of sub-clinically infected non-human reservoirs and/or vectors, such as migratory wild birds or imported ticks, might be plausible routes of the international spread of SFTSVs [27]. Consequently, non-human reservoirs and/or vectors would likely be critical sentinels to rapidly capture the introduction of nonendemic SFTSVs among the three countries.

Using the complete genomic sequences of SFTSVs, our phylogenetic analyses found the heterogeneity in the evolutionary characteristics of SFTSVs by gene segments. Longer between-genotype branches than within-genotype branches in our phylogenies likely explained that the between-genotype genetic variation would be much higher than within-genotype variation, especially in the M gene, and the accumulation of unusual within-genotype mutations would be limited compared to the between-genotype (or genotype-defining) mutations. Subsequently, we estimated the lower evolutionary rates and older divergence times of endemic SFTSVs in three countries than those in Fu et al [27], especially in the L and M genes. The ancestral SFTSV appeared to diverge into the C1 and C2 lineages around 500 years (L gene) or 900 (M gene) ago and the C2 lineage subsequently diverged into Japanese and Chinese—Korean endemic genotype B sub-lineages around 450 years ago (L and M genes) (Fig 2). However, we would carefully interpret the genealogy because highly heterogeneous evolutionary rates by gene regions (e.g., within-genotype vs between-genotype mutations) within the L and M genes might have lowered the average evolutionary rates.

Our selection profiles found that all three gene segments under intense purifying selection (dN/dS ratios = 0.03 to 0.13), which is close to the estimates of flaviviruses with one of the lowest dN/dS ratios (0.01 to 0.14) among RNA viruses [49]. It might indicate that most nonsynonymous mutations were deleterious, and the key mutations were very limitedly fixed in all three gene segments [49,50]. Furthermore, nonendemic SFTSVs generally disappeared after the introduction into other geographical regions failing to generate their own subclade, which possibly explained that the genetic turnover by the introduction of nonendemic SFTSVs would also be very limited. The genetic reassortments also appeared to be deleterious for SFTSVs. Our study observed genetic reassortment events in less than 5% of our SFTSV samples which was a similar result to the previous study (6.3%) [27]. The L-S and M-S reassortant strains widely switched their S gene segment with other genotypes, but all these strains failed to fix the reassortment trait and rapidly disappeared. The L-M reassortant strains appeared to form their own subclade, but they showed narrow reassortment compatibility because the L-M reassortant strains were limitedly observed between two genetically close subclades within the same genotype. Consequently, the epidemiological changes of human SFTS in three countries since 2010 (Fig 1) might not be followed by the emergence of novel SFTSV variants nor a substantial evolutionary change considering similar genealogy of SFTSVs by genotypes and evidence of intense purifying selection. On the other hand, the change of transmission dynamics of non-human reservoirs and/or vectors, such as tick and wildlife ecology or environmental condition through climate change [51], or the better SFTS detection, control and preventive measures might be more plausible factors to promote the current changes of human SFTS epidemiology.

Under intense purifying selection in the SFTSV evolution, our study found a few positive selection residues. The Gn region in the M gene harbored the largest number of amino acid residues under positive selection (residues 18, 151, 279, and 304) and showed the second highest non-synonymous substitution rate among six genetic regions. In addition, the Gc region in the M gene had two amino acid residues under positive selection (residues 77 and 398) and one fixed amino acid substitution through the L-M reassortment event (Q297P). Notably, the Gn and Gc regions in the M gene encode two components of the surface protein for viral adsorption and entry [52] and possibly played a key role to maintain the genetic diversity of SFTSVs. The recent study of the Gn region and human neutralizing antibodies revealed that domain III of the Gn head part is a critical region recognized by the host’s antibodies [53]. Interestingly, half (2/4) of the positively selected sites in the Gn localized in domain III (residues 249–313), probably indicating the action of selective pressure on the such immunodominant region, like other phleboviral Gn protein [54]. The NS region in the S gene had a relatively higher dN/dS ratio (0.13) and harbored fewer amino acid residues under negative selection (61.77%) than the other gene regions (Table 2). This finding agrees with the study of Lam *et al*. [55], suggesting that the NS region possibly be a preferential target for positive selection because the NS protein suppresses host antiviral immunity through the type I interferon (IFN) system in SFTSV’s infection [56]. Generally, infectious pathogens are forced to compete for their fitness among multiple strains [57,58]. However, SFTSV maintained genetic diversity through the cocirculation of multiple lineages over the evolutionary history with very limited extinction of genotypes [54]. Most vector-borne viral diseases, such as dengue or West Nile virus, typically promoted the cocirculation of multiple lineages through antibody-dependent enhancement (ADE) [59]. Despite limited reports of ADE by re-infection compared to other vector-borne diseases, our study suspected that ADE possibly drove to maintain the genetic diversity and allow cocirculation of multiple lineages [60].

Although we conducted a large-scale phylogenetic study to investigate the evolutionary characteristics of SFTSVs, we still acknowledge the importance of systematic data collection and the development of statistical approaches. Although our study efficiently collected more genetic sequences of SFTSV from the BV-BRC than the previous study [27], we could additionally obtain genetic sequences from the previous research publication (e.g., human SFTSVs from the outbreak in Japan from 2013 to 2014) [30]. The development of genetic resources repository systematically collecting genetic sequences from research publications as well as voluntary submission will improve the efficiency of genetic data collection and help to minimize geographical and/or temporal sampling bias in the phylogenetic estimation. In addition, the local selection pressure profiles might be inconsistently measured due to statistical sensitivity, genetic diversity of alignment, and length of gene segments [29,42,49,55,61]. Our study was also uncertain about how complex genetic mutations such as gene recombination influenced the SFTSV evolution [55,62,63]. In addition, the present study could not explore how key substitutions in the amino acid residues under positive selection functionally influenced the molecular evolution of SFTSVs. We expected that the reverse genetics incorporating serological approaches enables clarification of the implication of site-specific positive selection profiles in the immune-related genes (i.e., the NS and Gn/Gc), as demonstrated in other RNA viruses [64,65].

Our large-scale re-evaluation of SFTSV evolution facilitates step-changes in our understanding of SFTSV evolution and epidemiology through large-scale phylogenetic analyses using complete genome sequences. The origin of SFTSVs between China, Japan, and South Korea appeared to be far more ancient than previously known by maintaining their own geographical heterogeneity with infrequent international spread through non-human reservoirs and/or vectors. Furthermore, we characterized how purifying and positive selection profiles shaped the evolutionary dynamics of SFTSVs. Considering all, this study provides meaningful insight to better monitor and control the current cases of human SFTSV infection and to prepare for a potential subsequent generation of human-adaptive variants.

## Supporting information

S1 Fig Phylogenetic relationship among human, tick and non-human mammal host of SFTSV by (A) L, (B) M, and (C) S gene segments. In the MCC trees, tick and non-human mammal SFTSVs are labelled with red stars and blue squares. Human SFTSVs have no labels.
(TIF)

S1 Table The number of SFTSV sequences by the gene segment, collection country and host species. (DOCX)

S2 Table The complete list of reassortment events between each pair of Long (L), Medium (M) and Short (S) gene segment of SFTSV. (DOCX)

S1 Data The complete genome sequence sets (L, M, and S genes) used in this study. (ZIP)
