# Supplementary figures and images for: Genome-informed investigation of the molecular evolution and genetic reassortment of severe fever with thrombocytopenia syndrome virus
Source: PLoS Negl Trop Dis. 2023 Sep 15;17(9):e0011630. doi: 10.1371/journal.pntd.0011630 (PMC10529592; doi:10.1371/journal.pntd.0011630)

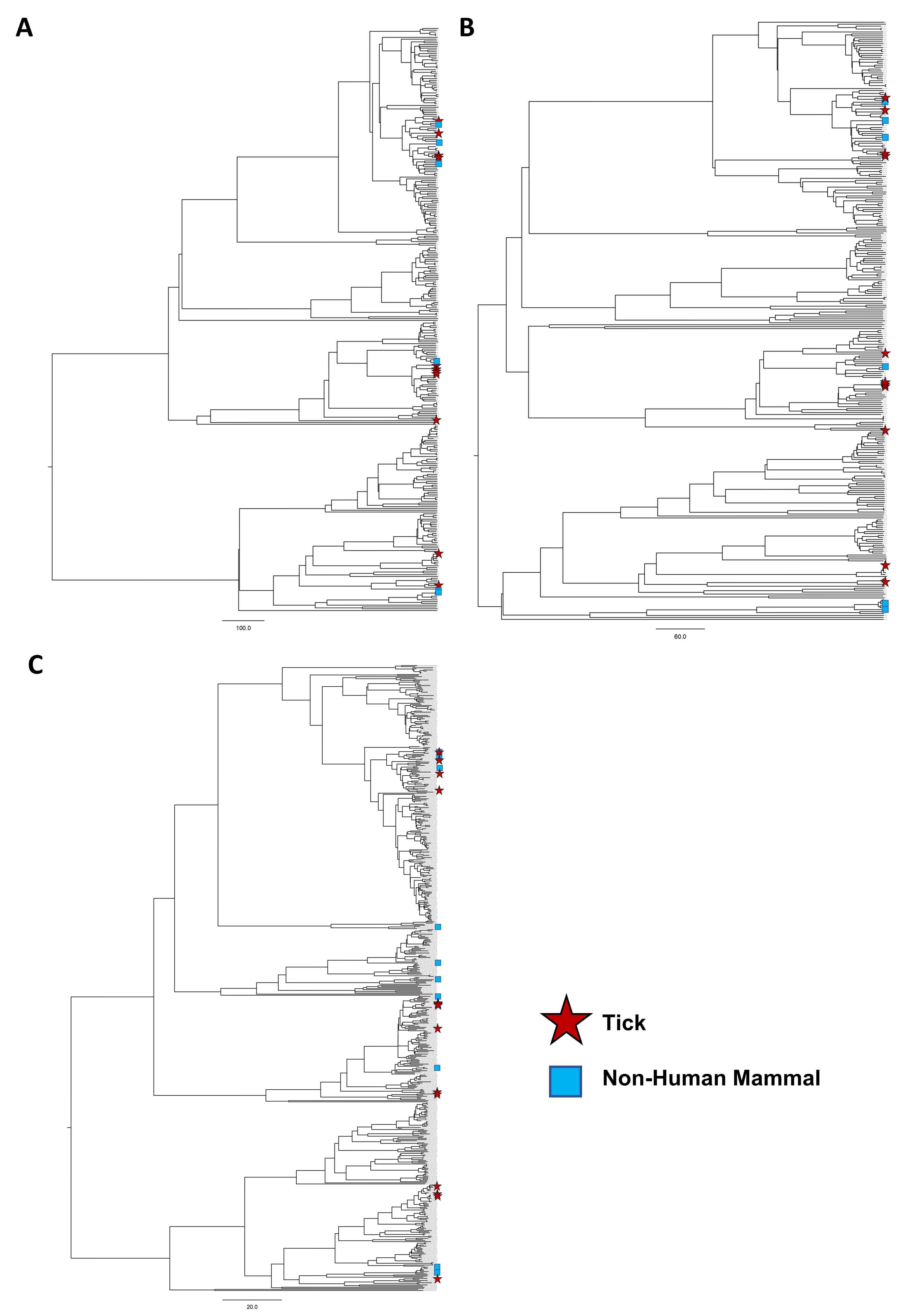

Supplement: S1 Fig — In the MCC trees, tick and non-human mammal SFTSVs are labelled with red stars and blue squares. Human SFTSVs have no labels. (TIF) [file pntd.0011630.s001.tif]
