# Supplementary material for: Genome-informed investigation of the molecular evolution and genetic reassortment of severe fever with thrombocytopenia syndrome virus
Source: PLoS Negl Trop Dis. 2023 Sep 15;17(9):e0011630. doi: 10.1371/journal.pntd.0011630 (PMC10529592; doi:10.1371/journal.pntd.0011630)
Supplement: S1 Table — (DOCX) [file pntd.0011630.s002.docx]

**S1 Table. The number of SFTSV sequences by the gene segment, collection country and host species.**

| **Gene** | **Country** | **Host species** | **Number of sequences** |
| --- | --- | --- | --- |
| **L** | China | Human | 217 |
|  |  | Tick | 11 |
|  |  | Non-human mammal | 6 |
|  |  | Unknown | 6 |
|  | Japan | Human | 46 |
|  | South Korea | Human | 12 |
|  |  | Tick | 1 |
|  |  | Unknown | 8 |
|  | Sub-total | | 307 |
| **M** | China | Human | 222 |
|  |  | Tick | 10 |
|  |  | Non-human mammal | 6 |
|  |  | Unknown | 13 |
|  | Japan | Human | 43 |
|  | South Korea | Human | 23 |
|  |  | Tick | 1 |
|  |  | Unknown | 8 |
|  | Sub-total | | 326 |
| **S** | China | Human | 384 |
|  |  | Tick | 11 |
|  |  | Non-human mammal | 10 |
|  |  | Unknown | 19 |
|  | Japan | Human | 64 |
|  | South Korea | Human | 42 |
|  |  | Tick | 3 |
|  |  | Unknown | 31 |
|  | Sub-total | | 564 |
| **Total** | | | 1197 |
