# Supplementary material for: Genome-informed investigation of the molecular evolution and genetic reassortment of severe fever with thrombocytopenia syndrome virus
Source: PLoS Negl Trop Dis. 2023 Sep 15;17(9):e0011630. doi: 10.1371/journal.pntd.0011630 (PMC10529592; doi:10.1371/journal.pntd.0011630)
Supplement: S2 Table — (DOCX) [file pntd.0011630.s003.docx]

**S2 Table. The complete list of reassortment events between each pair of Long (L), Medium (M) and Short (S) gene segment of SFTSV**

| **Year** | **Country** | **Candidate Strain** | **Gene reassortment pair** | | |
| --- | --- | --- | --- | --- | --- |
|  |  |  | **L-M** | **L-S** | **M-S** |
| 2011 | China | China/Human/2011YSC60/2011 |  |  | Yes |
| 2011 | China | China/Human/YSC3/2011 |  | Yes | Yes |
| 2011 | China | China/Human/YXX2/2011 |  | Yes |  |
| 2012 | China | China/Human/HNXY/182/2012/08 |  | Yes |  |
| 2012 | China | China/Human/HNXY/31/2012/08 |  | Yes |  |
| 2012 | China | China/Human/LN2012/58/2012 |  | Yes |  |
| 2012 | China | China/Human/ZJZHSH/HCY/China/06/2012 | Yes |  |  |
| 2012 | China | China/Human/ZJZHSH/SHZ/China/05/2012 | Yes |  |  |
| 2012 | China | China/Human/ZJZHSH/XAM/China/06/2012 | Yes |  |  |
| 2012 | China | China/Human/ZJZHSH/ZLD/China/06/2012 | Yes |  |  |
| 2012 | China | China/Human/ZJZHSH/ZLN/China/06/2012 | Yes |  |  |
| 2013 | China | China/Human/DS02/CHN/2013 | Yes |  |  |
| 2013 | China | China/Human/NB32/CHN/2013 |  |  | Yes |
| 2013 | China | China/Human/NB38/CHN/2013 |  | Yes | Yes |
| 2014 | China | China/Human/JS2014/39/2014 |  | Yes | Yes |
| 2014 | China | China/Human/ZJZHSH/LWL/China/08/2014 | Yes |  |  |
| 2016 | China | China/Human/HB2016/013/2016 |  | Yes | Yes |
| 2016 | China | China/Human/HB2016/034/2016 |  | Yes |  |
